# One hundred years of neurosciences in the arts and humanities, a bibliometric review

**DOI:** 10.1186/s13010-023-00147-3

**Published:** 2023-11-09

**Authors:** Manuel Cebral-Loureda, Jorge Sanabria-Z, Mauricio A. Ramírez-Moreno, Irina Kaminsky-Castillo

**Affiliations:** 1 Humanistic Studies Department, School of Humanities and Education, Tecnologico de Monterrey, Monterrey, Mexico; 2 Institute for the Future of Education, Tecnologico de Monterrey, Monterrey, Mexico; 3 Mechatronics Department, School of Engineering and Sciences, Tecnologico de Monterrey, Monterrey, Mexico

**Keywords:** Neuro-Arts, Neurosciences, Humanities, Scopus, R programming, Neuroaesthetics, Higher Education, Educational innovation

## Abstract

**Background:**

Neuroscientific approaches have historically triggered changes in the conception of creativity and artistic experience, which can be revealed by noting the intersection of these fields of study in terms of variables such as global trends, methodologies, objects of study, or application of new technologies; however, these neuroscientific approaches are still often considered as disciplines detached from the arts and humanities. In this light, the question arises as to what evidence the history of neurotechnologies provides at the intersection of creativity and aesthetic experience.

**Methods:**

We conducted a century-long bibliometric analysis of key parameters in multidisciplinary studies published in the Scopus database. Screening techniques based on the PRISMA method and advanced data analysis techniques were applied to 3612 documents metadata from the years 1922 to 2022. We made graphical representations of the results applying algorithmic and clusterization processes to keywords and authors relationships.

**Results:**

From the analyses, we found a) a shift from a personality-focus quantitative analysis to a field-focus qualitative approach, considering topics such as art, perception, aesthetics and beauty; b) The locus of interest in fMRI-supported neuroanatomy has been shifting toward EEG technologies and models based on machine learning and deep learning in recent years; c) four main clusters were identified in the study approaches: humanistic, creative, neuroaesthetic and medical; d) the neuroaesthetics cluster is the most central and relevant, mediating between creativity and neuroscience; e) neuroaesthetics and neuroethics are two of the neologism that better characterizes the challenges that this convergence of studies will have in the next years.

**Conclusions:**

Through a longitudinal analysis, we evidenced the great influence that neuroscience is having on the thematic direction of the arts and humanities. The perspective presented shows how this field is being consolidated and helps to define it as a new opportunity of great potential for future researchers.

## Introduction

The interaction between neuroscience and arts and humanities has become a prolific area of research that has increasingly gained attention. From the end of the century, when the necessity of a solid neurological science of art is claimed [41], throughout works using more technical devices to study creativity [18, 19], there can be found nowadays many works that use directly the single word neuroaesthetics applied to specific humanistic areas, such as poetry (Jacobs, 2017, painting [33], sculpture [47], music [23], or dance [53].

These kinds of research are concerned about the status of the mind, and how artistic practices can help to understand, or even improve, our cognitive performance. More concretely, some studies are focussed on how neurological damages affect, or not, artistic experiences [13, 31], meanwhile others are more dedicated to analyzing aesthetics in healthy individuals [17, 36], and even to promote well being [35]. For our proposal, all these tendencies are very related, due their inspiration on an humanistic understanding of the brain, expliciting a convergent area or research.

To address this compounded landscape, a bibliometric approach is suggested, in order to make explicit its evolution and the internal organization. A bibliometric literature review studies the quantitative aspects of large sets of academic literature. Since it is based on statistical, mathematical, and methodological techniques, it reduces many of the weaknesses of conventional review methods such as position bias and limited textual query modes. This makes it an appropriate method to amplify the knowledge and understanding of growing fields [43]. Bibliometrics is therefore a very relevant and useful method to track, measure and analyze burgeoning amounts of bibliographic data, and well supported by newer and more accessible statistical and computational tools [40].

In the case of neurosciences, some previous studies perform similar methods. Illes et al. [24] wrote a brief letter where they analyze the historical and emerging trends in neuroimaging. They found that both the number of articles as well as journals had significantly grown from the 1990s to early 2000. A more complete study appeared in 2017 [51], analyzing 340,210 publications in the period between 2006 and 2015. They develop an interesting set of metrics and visualizations, such as term maps, annual high-impact terms, and changes in research areas,but they do not identify arts and humanities among the twelve most relevant knowledge areas. A similar article, published in the same year and conducted by the same author [52], identifies the 100 most cited articles in neuroscience, and classifies them in 4 topics: neurological disorders, prefrontal cortex and associated emotion, brain network, and brain mapping,none of them are oriented to artistic and humanistic experiences. More recently, Ismail & Karwowski [26] propose a suggestive bibliometric study, this time combined with literature review methods, and focussing on EEG quantification research for human cognitive performance.

However, what remains unanalyzed from a bibliometric point of view is the specific relationship between neuroscientific approaches and the artistic and humanistic experiences, evaluating the impact that the studies about the brain have got in the humanities discipline, and if it can be useful to understand this neologism of recent creation which is neuroaesthetics. Certainly, Anglada-Tort & Skov [2] have already worked in a large review with more than 20,000 articles, but dealing in a general way with the relationship of all kinds of sciences with the term aesthetics, asking about its meaning for scientific production, and evidencing a certain lack of unity, common problems and strategies. That is why Skov & Nadal (2020, 2023) propose to separate aesthetics studies of neuroaesthetics, pointing out that there is an interest in neuroscientific studies of aesthetics that has to do with marketing and consumer experience, and not with art or even less humanities. Just because of this, it is pertinent to research to what degree humanities as a discipline can effectively adopt alien approaches such as those produced by neuroscience [9, 48].

Drawing on the intellectual intersection of these topics, the following research questions were posed, in accordance with the approach observed in studies of this nature, studies conducted by the authors, and discussions with experts in the field. The authors' personal experience in educational and research settings related to experimentation in advanced research laboratories was also considered. Moreover, providing new contributions to research in neurotechnologies was a strong driver for the themes generated and the underlying research questions (see Table 1).

**Table 1:** Themes and research questions that guided the study

Themes	Research questions (RQ)
Scientific production exploration	**RQ1.** What is the relevance of the convergence of neuroscience and arts & humanities as a field of interest for the researchers?
	**RQ2.** How has this convergence been built in terms of historical periods?
Specific relationships among keywords related to neuroscience in arts & humanities	**RQ3.** Which are the most influential keyword trends when considering neuroscience applied to arts & humanities?
	**RQ4.** How are these trends distributed in terms of relevance and centrality measures?
	**RQ5.** What clusters can be distinguished within the most frequent keywords network and which would be their most relevant nodes?
Main theoretical approaches based on influential papers	**RQ6.** What are the most influential papers and how do they relate in terms of network analysis?
	**RQ7.** What are the main clusters in this network and what do they tell us about the different theoretical approaches?
Fields of study application	**RQ8.** How consolidated is this convergence of studies as a knowledge area?
	**RQ9.** What are the main issues and controversies this convergence faces?
	**RQ10.** What technologies have been implemented along its historical evolution?
Future studies	**RQ11.** What are the expected trends that may dominate in the coming years in this convergence of this area of study?

## Methods

Data for the present study was retrieved from the Scopus academic abstract and citation database in December 2022; although other databases are available, Scopus is the most recommended for quantitative analysis of scientific literature, since it guarantees the highest quality data indexed [4]. A search descriptor covering the main topics of interest was agreed on by the authors, intended to include combinations of compound words and constructs in a range close to a century. To achieve this, the search consisted of the combination of the word “neuro*”, with an asterisk, in order to encompass all words with this prefix, and the words “arts”, “humanities”, “human sciences” and “artistic” -using an OR operand to look for each individual combination-, as can be observed in the following script.

( TITLE-ABS-KEY ( neuro*) AND TITLE-ABS-KEY ( {arts} OR humanities OR "human sciences" OR artistic).

This search yielded a set of 3860 documents; however, part of the results of this search were related to the field of "martial arts", which we consider to be a term rather linked to sports, and therefore far from the academic concept of arts associated with the humanities. Thus, we decided to exclude the term "martial arts" from the search string, as shown below, also acknowledging that its presence implied an isolated cluster in the findings. No other similar constructions were identified that would have a significant impact.

( TITLE-ABS-KEY ( neuro*) AND TITLE-ABS-KEY ( {arts} OR humanities OR "human sciences" OR artistic) AND NOT TITLE-ABS-KEY ( "martial arts")).

**Fig. 1:**
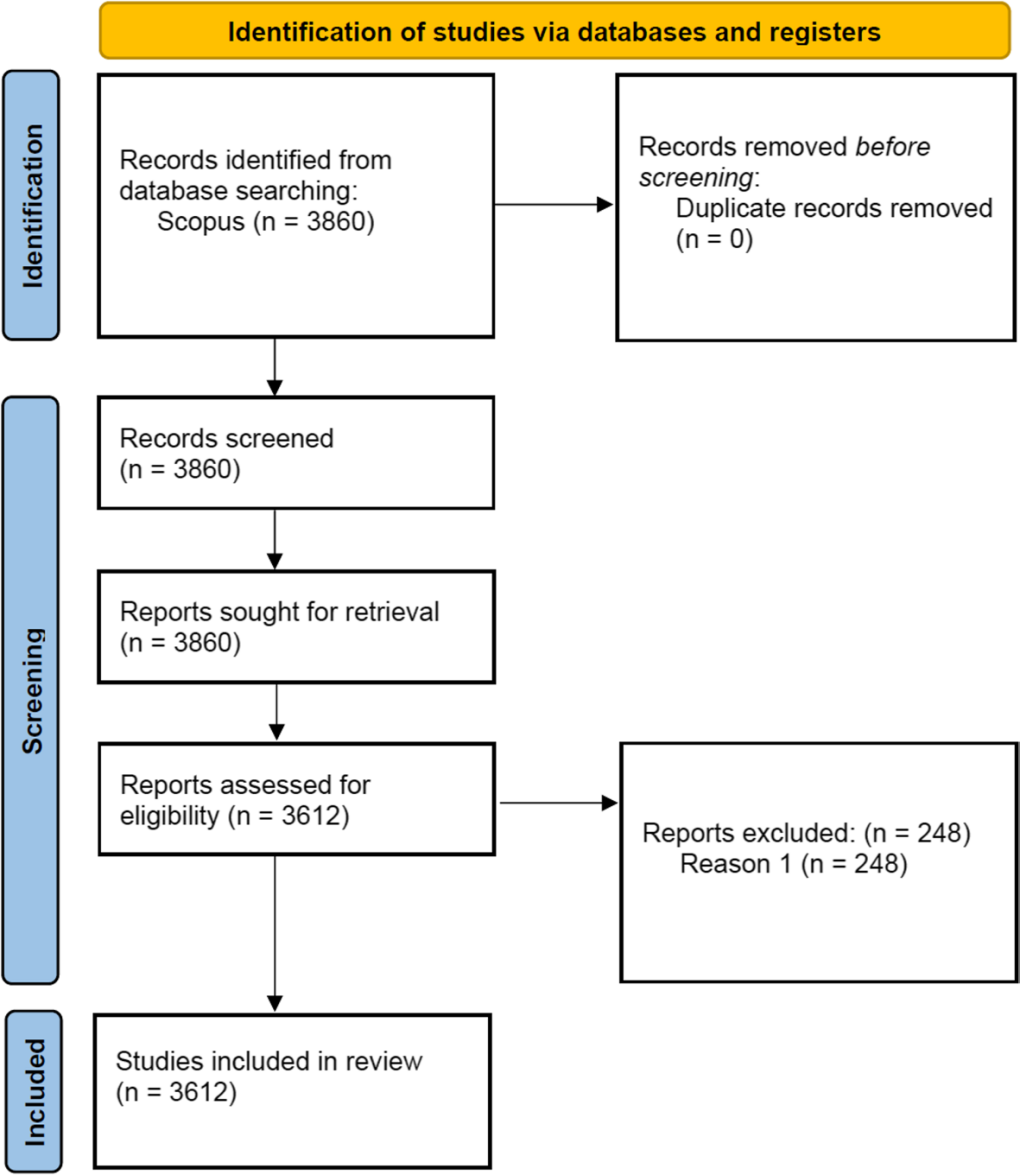
PRISMA diagram of literature search implemented. Items were excluded if they contained the terms “martial arts” (Reason 1)

Under these new settings, a total of 3612 documents were found, and after revision, these were the ones considered in this work, as can be observed in Fig. 1.

In addition to its impact, the SCOPUS database was chosen because of its compatibility to insert filters and graphs using R, a widely accepted language for computing graphics. R programming was the primary tool employed for the data analysis, combined with the use of additional packages: *bibliometrix* [3], *widyr*, [42] and *tidyverse* [50]. These packages permit the development of various analysis and visualizations, the calculation of correlations between keywords and the management and visualization of data, respectively. The Gephi software was also used for the application of network algorithms and for further visualization, in order to develop relationship networks [5].

Before the analysis, some previous work was performed, in order to clean the data, obtaining stronger references which, otherwise, could have been excluded by the algorithms. The applied transformations were: differents versions of neuroaesthetics -such as with or without hyphen- were unified as neuroaesthetics; the terms parkinson and parkinson’s disease or derivatives were unified as parkinson; all the different versions of EEG -such as electroencephalography, electroencephalogram or others, including specifications such as EEG laterality or EEG reactivity- were unified as EEG; different versions of fMRI -such as MRI or functional magnetic resonance imaging- were unified as fMRI. Finally, other terms with plural and singular derivatives were unified, choosing the singular expression -e.g. art/arts or neuroscience/neurosciences-.

**Fig. 2:**
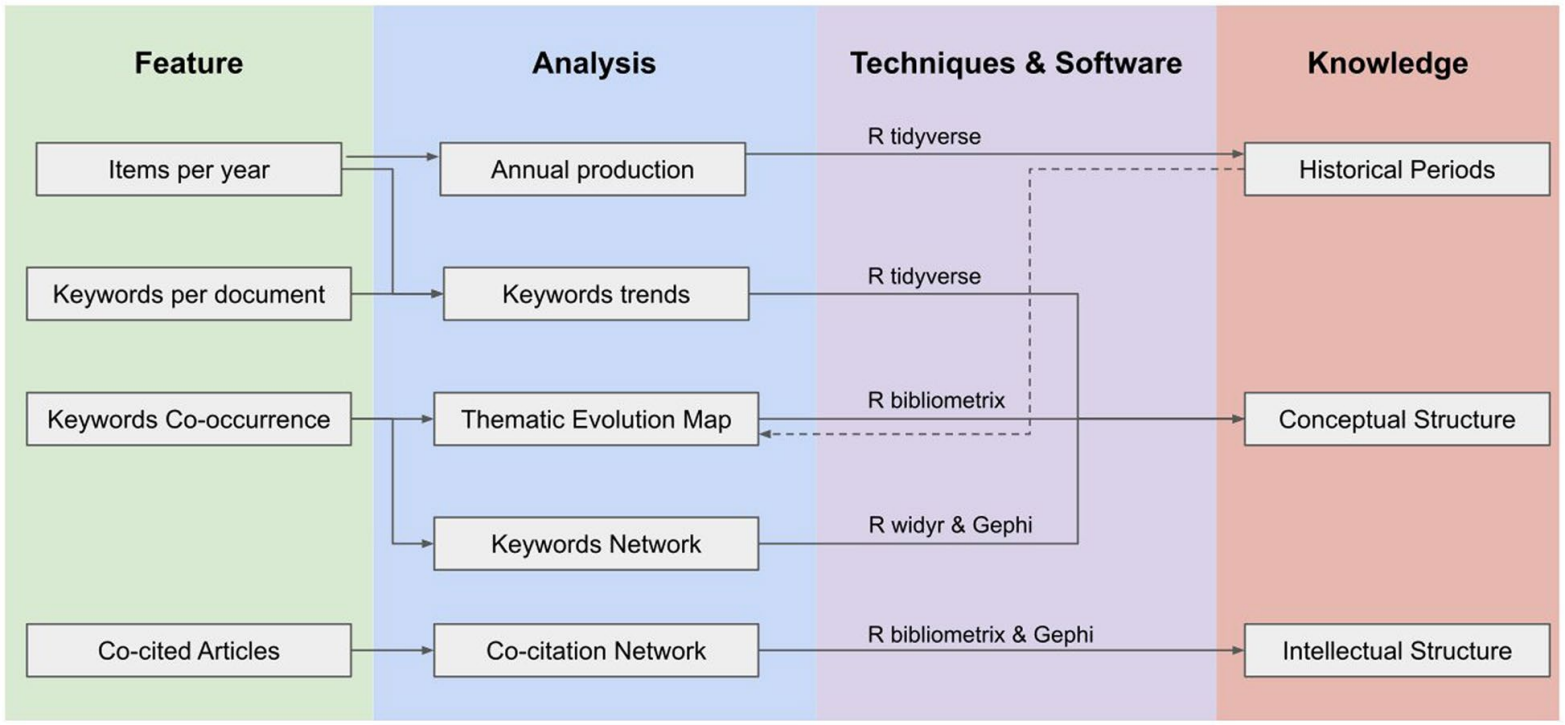
Representative diagram of the bibliometric process implemented in this work

Four main analyses were implemented with R software using all the items found in the SCOPUS database with the proposed string. The idea behind each bibliometric analysis is explained below, and a representative diagram of this process is presented in Fig. 2.

Firstly, an exploratory analysis of multiple levels of the found references (reference type, year of publication, common keywords, authors) was conducted. Then, a thematic evolution map was drawn to analyze the conceptual evolution of keyword co-occurrences. Clusters of references were mapped into a 2D Cartesian coordinate system (development and relevance degree), and grouped into four groups (Niche, Emerging, Motor and Basic themes), following Callon’s co-word analysis [8] as provided by the *thematicEvolution* function of the *bibliometrix* package. Such conceptual structure was complemented by a network analysis of the 250 most frequent keywords for the whole sample, without taking into account any period. This analysis was performed by using the *pairwise_count* function of the *widyr* package and the Gephi software, which clusterizes the network using the modularity algorithm. Finally, a similar network analysis was made regarding the intellectual structure, this time through the *biblioNetwork* function of the *bibliometrix* package, considering when two articles are cited together by a document of the sample. Such an approach generates the co-citation network that has also been clusterized by means of the modularity algorithm.

## Results

The literature found using the proposed string was analyzed in detail using the tools mentioned in the Methods Section.

Initially, an exploratory analysis was performed on all the found items, on four levels: (1) main information (timespan, reference source, documents, annual growth rate, document average age, average citations per document, and references); (2) authors (authors number, authors of single-authored documents, number of co-authors per document, and percentage of international co-authorships); (3) document type (article, book, book chapter, conference paper, conference review, editorial, erratum, letter, note, review, and short survey); and (4) document content (keywords plus ID, and authors’ keywords DE). The results of this exploratory analysis are presented in Table 2. They point out that it is a very productive area, with a positive annual growth rate and an impressive average citation per doc, obtaining a h-index of 110, more than the highest found in comparisons such as the one made by Malesios & Psarakis [34] for different scientific fields.

**Table 2:** Summary of literature found in this (NeuroArts) bibliometric study at four different levels: main information, authors, document type, and document content. The earliest document found in the range of a century backwards dates from 1934

MAIN INFORMATION ABOUT DATA		AUTHORS COLLABORATION	
Timespan	1934:2022	Single-authored docs	1540
Sources (Journals, Books, etc.)	2155	Co-Authors per Doc	2.88
Documents	3612	International co-authorships %	16.09
Annual Growth Rate %	6.5%	**DOCUMENT TYPES**	
Document Average Age	9.38	article	2083
Average citations per doc	17.63	book	181
H Index	110	book chapter	295
References	189,324	conference paper	335
**DOCUMENT CONTENTS**		conference review	12
Keywords Plus (ID)	14,152	editorial	47
Author's Keywords (DE)	8312	erratum	14
**AUTHORS**		letter	25
Authors	8999	note	59
Authors of single-authored docs	1337	retracted	1
		review	518
		short survey	41

**Fig. 3:**
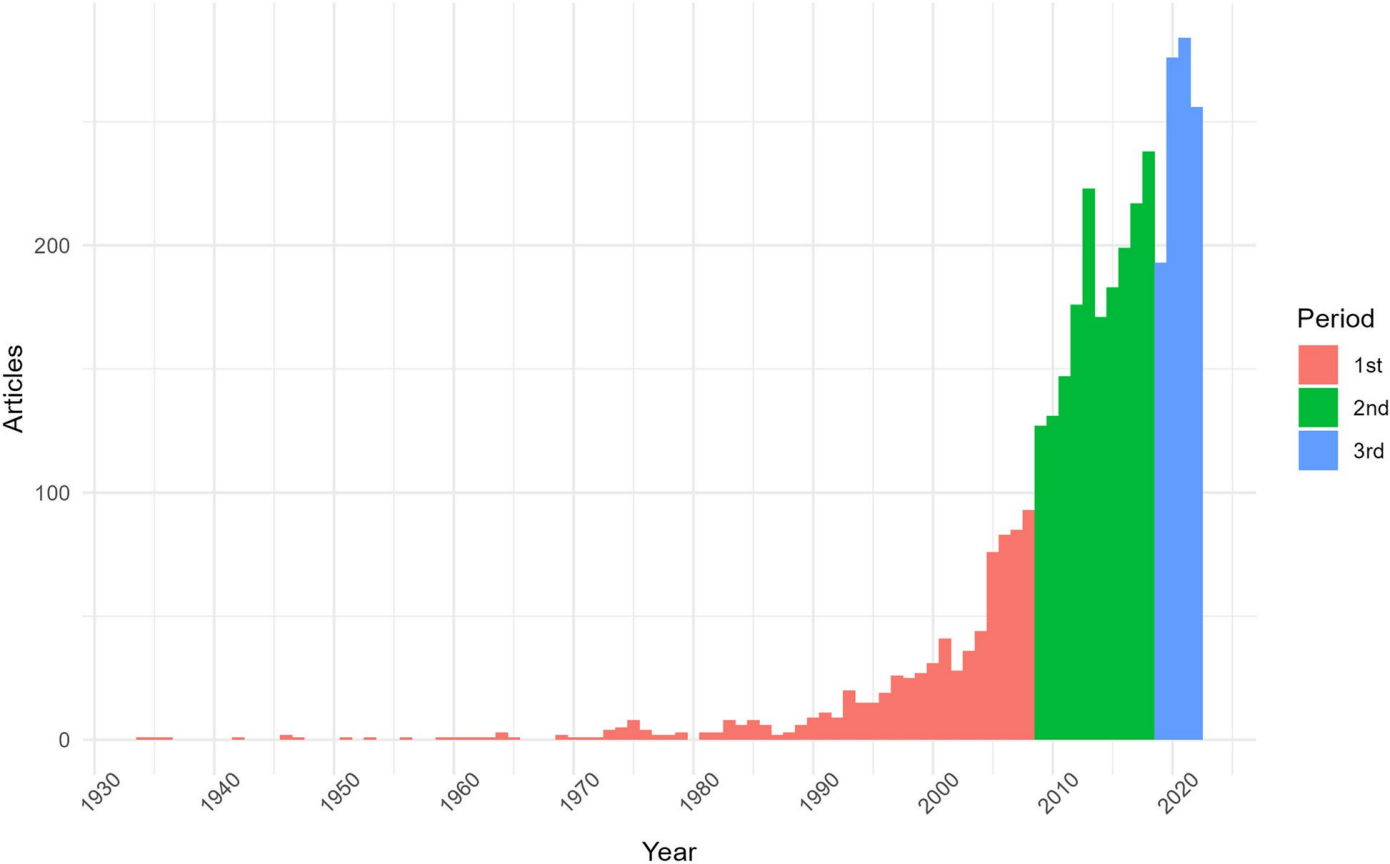
Distribution of published NeuroArts articles from 1934—2022 and the three identified publication periods: from the first document found in Scopus in 1934 to 2008; from 2009 to 2018; and from 2019 to 2022

Documents related to this bibliometric study were found from the 1934–2022 timespan. When visualizing the annual production -see Fig. 3, three different periods were found, taking into account the different peaks and its particular distribution at specific years within their timespans. These periods were identified as follows: a first period from 1934 to 2008, then considering an important jump in productivity occurred in 2009; a second period from 2009 to 2018, a year in which a production peaks. This then decreases considerably in 2019, with the final period being 2019 to 2022, when production recovers and provides the latest trends in the field. Clearly, despite some downgrades, the overall trend in production has been positive and even exponential, describing a curve rather than a straight line.

**Fig. 4:**
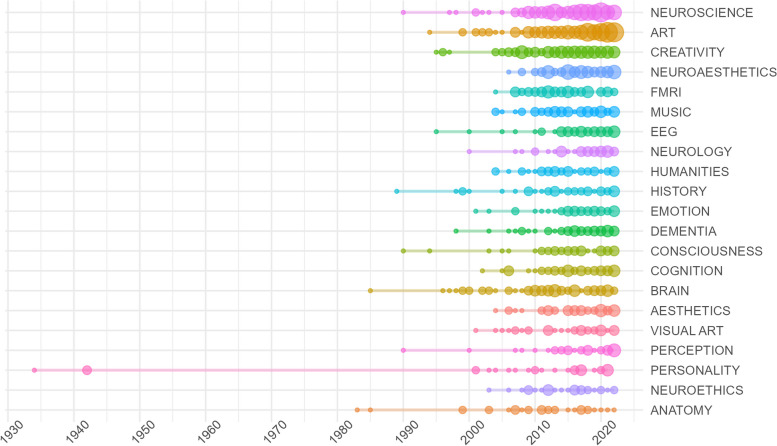
Twenty most frequent keywords trends ordered by their total frequency in the sample. The size of each point shows the number of appearances that each keyword has by year

Documents were also analyzed in terms of their keywords. Figure 4 shows the trend of the twenty most frequent keywords in the literature found by the bibliometric study, where a representation of the number of appearances per year of each keyword is shown. The first evidence is that the beginning of this convergence of studies occurs with the analysis of personality, far ahead of all other trends, followed by anatomy and the brain. From the other side, it is worth highlighting the prominent increase of the keyword art in the last five years, together with the concept of neuroaesthetics and the use of the EEG (more frequent in the last years than fMRI). Other current trends are the studies of emotion and perception.

**Fig. 5:**
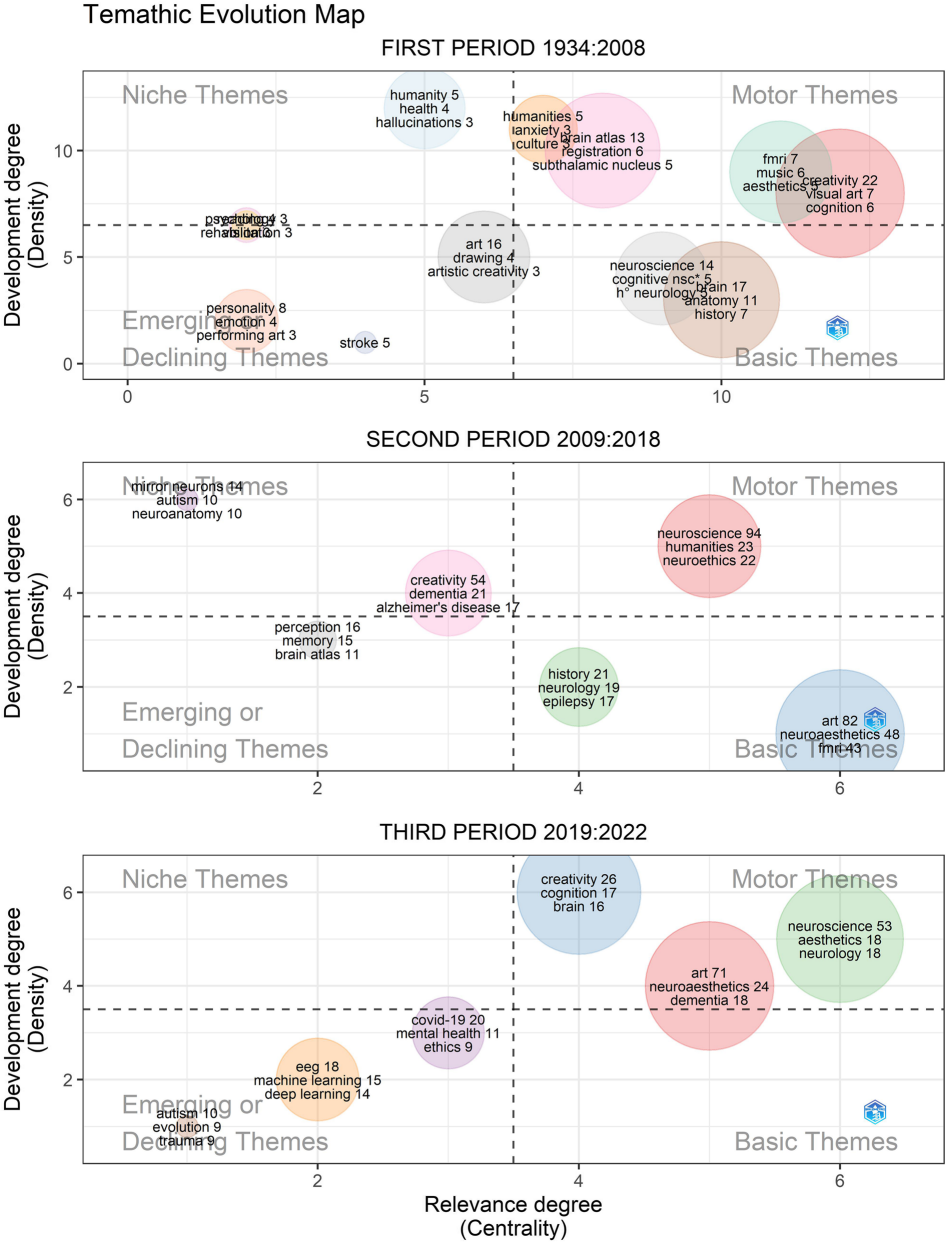
Thematic Evolution maps for the three time periods. Each map shows niche, motor, emerging and basic themes at each period. The 200 most frequent keywords were taken into account with a minimum of 5 times of frequency and a maximum of three labels by cluster

These trends can best be seen in the thematic evolution map in Fig. 5, divided into the three periods found above. Within the first one (1934 to 2008), brain and anatomy are the basic themes, along with neuroscience and cognitive neuroscience; meanwhile, creativity stands out as a motor theme, together with the fMRI technique. For the second period (2009 to 2018) the themes are more concentrated. Neuroscience, which was a basic theme in the first period, becomes a motor theme, already accompanied by humanistic concepts, such as humanities and neuroethics; meanwhile, art, neuroaesthetics and fMRI appear as basic themes also with high levels of frequency. For the last period, the lack of basic themes is very significant, evidencing the dynamism of this field of studies: there are only motor themes and emerging or declining themes. Observing these last motor themes with more detail, all of them aggregate terms coming from neurosciences and humanities together, consolidating the convergence of neuroaesthetics as an integrated field of study. At the same time, new techniques involving EEG, machine learning and deep learning become emerging themes, as well as the question of mental health during Covid-19 pandemic.

**Fig. 6:**
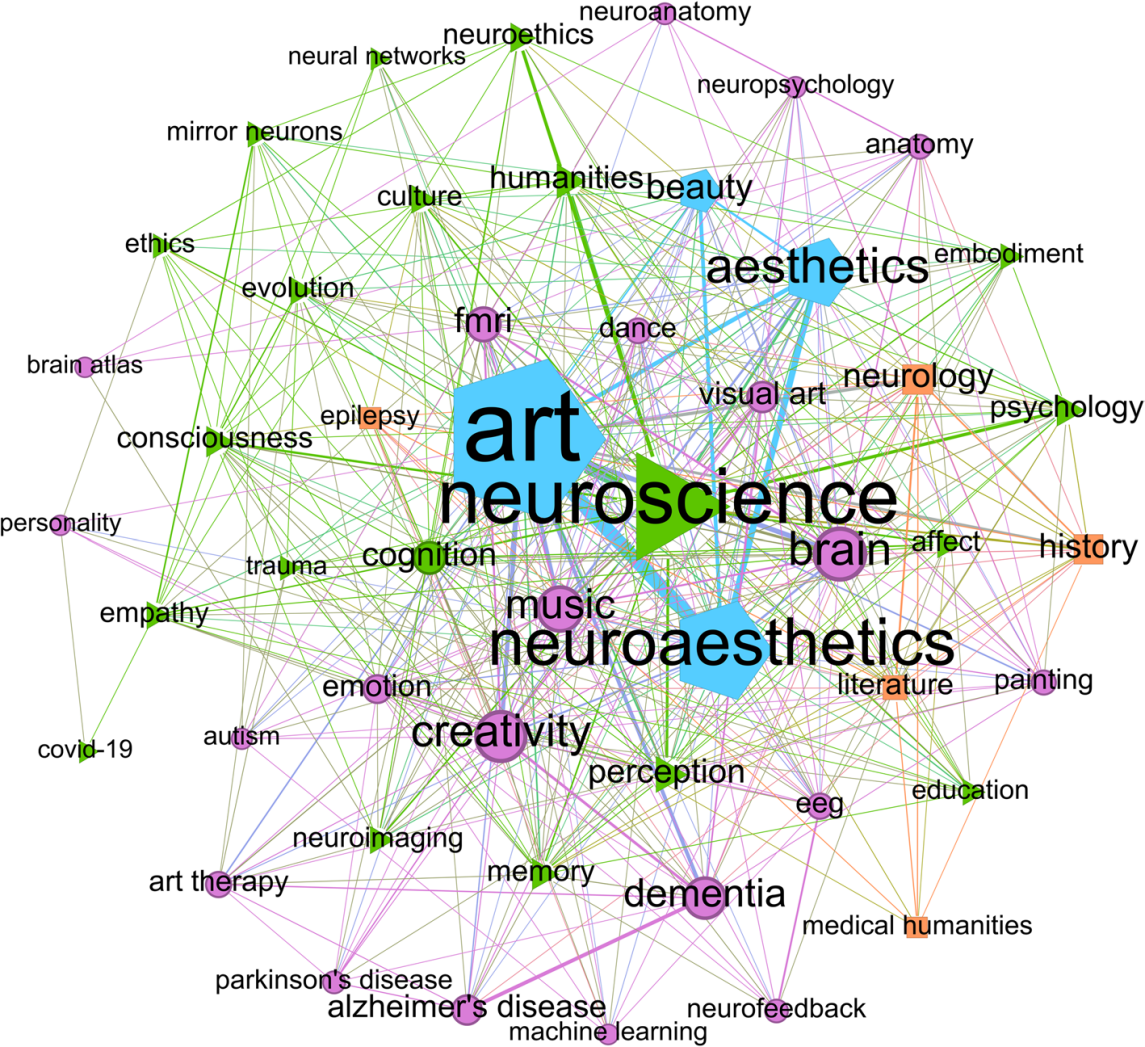
50 Most frequent co-occurring author keywords clustered using the modularity algorithm. The size of the nodes, as well as the size of the labels, was set according to their weighted degree. Clusters were represented with color and polygonal shapes

Figure 6 shows a representation of co-occurrence of keywords across this bibliometric study. By using the modularity algorithm, the 50 Most frequent co-occurring keywords were clustered into four groups. Each cluster is formed by a set of nodes, related to each other within the same cluster, where the size of each node reflects the co-occurrence frequency of such keywords. Find below a definition of each cluster.

Green triangular cluster: neuroscience in a more humanist sense, focused on consciousness, mind and epistemology, with terms such as humanities, psychology, embodiment, empathy, education or neuroethics. This cluster also includes technical aspects related to cognition and perception, with neuroimaging being the technology that is most closely linked to this cluster, as the tool to measure brain activity within medical spaces.

Purple circular cluster: it is focused on the creative and artistic side of neuroscience, but it also includes different diseases, such as dementia, Alzheimer's, or Parkinson's, as well as their modes of therapy and rehabilitation. It is interesting because the intersection of both concepts reflect questions that researchers have been asking in the neuroarts field, such as: How can art help heal? How do people with this type of problem react to creative processes? Can art be an alternative to conventional communication? How does damage to a region of the brain affect artistic expression and composition? The arts most linked to these questions, as shown by the cluster, are painting, music and dance.

Blue pentagonal cluster: It is the smallest cluster, but the densest and most important in terms of its weights, as can be seen by the size of each node. This shows that there is a close and strong interconnection between its nodes, which are focused on art, accompanied by the terms neuroaesthetics, aesthetics and beauty, so it can be called the neuroaesthetic one. It is a cluster, in turn, highly interconnected with the cluster based on neuroscience and the creative cluster, located in the middle of both.

Finally, the orange square cluster is the least relevant and most isolated, focused on medical approaches to neurology, including terms such as literature, and showing how there are specific studies of language and its correlation with anatomical areas of the brain.

**Fig. 7:**
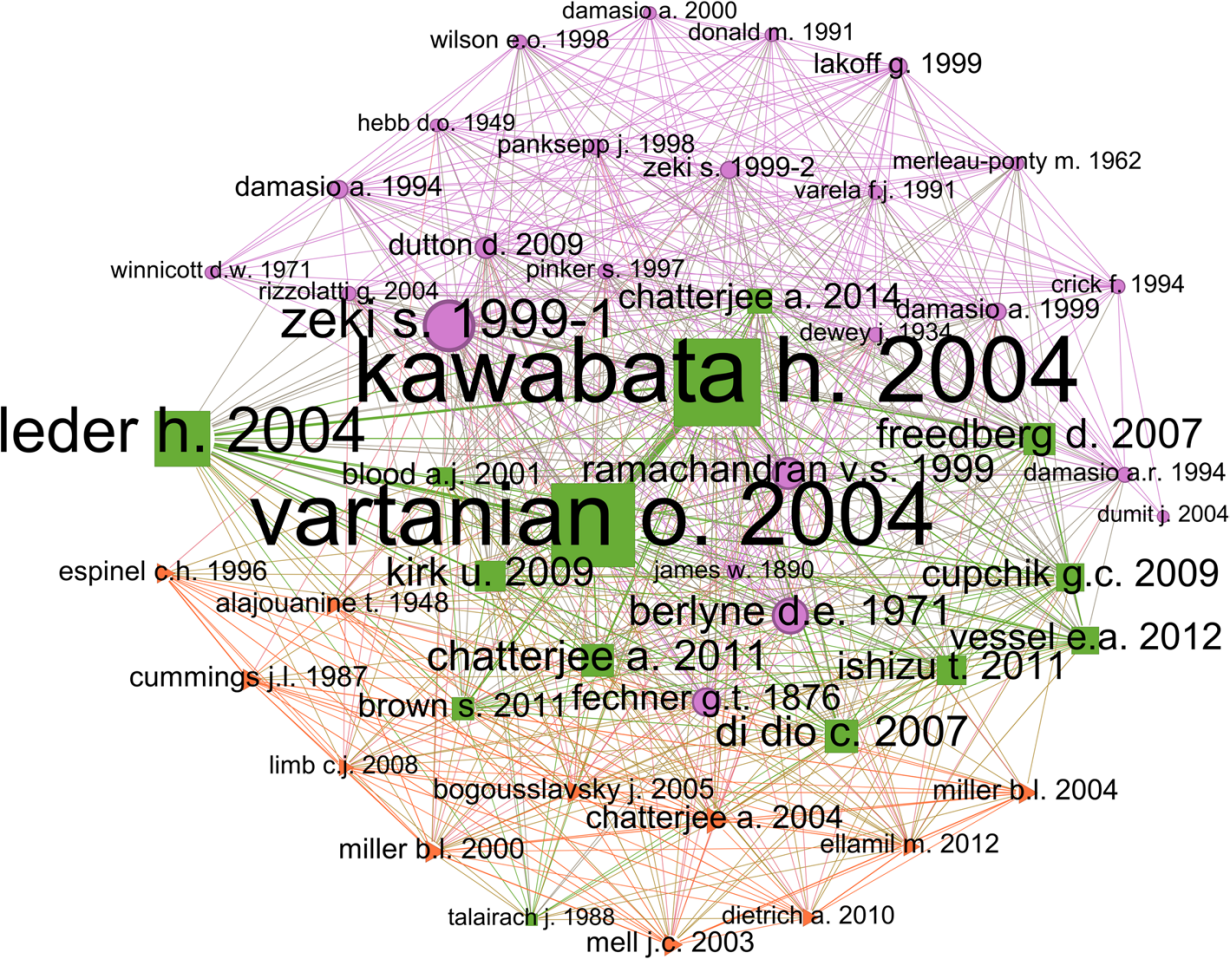
50 most frequent co-cited articles clustered using the modularity algorithm. The size of nodes and labels was set according to their weighted degree. Clusters were represented with color and polygonal shapes

Similar to Figs. 6 and 7 shows a representation of the most co-cited articles, within this bibliometric study. The 50 most co-cited articles were clustered into three main groups. Each cluster is formed by a set of nodes, connected to articles which are co-cited, within the same cluster, or to other clusters; and the size of each node and label reflect the amount of times an article is co-cited. Therefore, bigger nodes and labels represent the most co-cited articles.

Green square cluster: The most relevant paper of the whole network is *Neural Correlates of Beauty* [29], where the authors demonstrate, using fMRI, how beauty in painting is received by the brain, stimulating different areas regardless of a more beautiful or ugly painting, independent of style. The second most relevant paper, also identified within the same cluster, is *Neuroanatomical correlates of aesthetic preference for paintings* [49], where the authors show, again using fMRI, how differential patterns of brain activation rise in response to painting aesthetic preference. A third paper from 2004 occupies the third position within the network regarding their weighted degree: *A model of aesthetic appreciation and aesthetic judgments*, where Leder et al. [32] develop an information-processing stage model of art experience. They suggest 5 stages of reception -perception, explicit and implicit classification, mastering, and evaluation, together with two outputs,emotion and judgements. These three papers of the green square cluster were written in the same year, 2004, and all evaluating the cognitive reaction to art experience.

Other references that hold high weighted degree within the green square cluster are: *Artistic Production Following Brain Damage: A Study of Three Artists* [12] that penetrates neurological bases of art production comparing the influence of each hemisphere,the book *Aesthetics and psychobiology* [6], a classical reference of the field, where the author claims for the role of experiments in psychology of the aesthetics, far away from the Freudian non-empirical approaches,the paper of Ishizu & Zeki [25] *Toward A Brain-Based Theory of Beauty* where in an experiment with 21 people they prove the correlation between brain area activity and the experience of beauty derived from different sources; *Motion, emotion and empathy in esthetic experience* [22], where the authors expose the role of mirroring embodied mechanisms that respond empathetically to visual art and images in general,or *The golden beauty: brain response to classical and renaissance sculptures* [16], which also uses fMRI techniques.

Examining the purple circular cluster, it highlights the work of Zeki & Bartels [54] *Toward a Theory of Visual Consciousness*, which studies the visual brain processing systems, and their interconnectivity; another paper that can be pointed out is *The science of art: A neurological theory of aesthetic experience* [41], which is a theory of human artistic experience describing the neural cognitive circuit involved,as well as the book *Philosophy in the flesh: The embodied mind and its challenge to Western thought* (Lakoff & Johnson, 1999), a classical reference in the analytic philosophy where the authors claim an embodied reason and a metaphorical thinking for a cognitive psychology of the unconscious.

Compared with the green square cluster, although both are dealing with the relation between brain and cognitive activity with art and aesthetic experiences, the last one is more focused in the mechanisms and the processes involved, rather than of the areas, and even with experiences that are not so artistic, where cognitive processes and visual interaction are more important. Also, it is in the purple circular cluster where the most classical references are present, including the works of Damasio -*Descartes' error* (1994); and *The feeling of what happens* (1999); as well as the work *The principles of psychology* (James, 1890), a key reference in the constitution of psychology as a science. These titles reinforce the idea of behavioral observation experiments which, although they have empirical foundations, analyses like fMRI are less employed.

There is a third cluster, the orange triangular cluster, with fewer nodes, and significantly less-weighted degrees. The highlighted papers of the cluster are: *Cosmetic neurology: The controversy over enhancing movement, mentation, and mood* [10], *Artistic Creativity, Style and Brain Disorders* [7], *Functional correlates of musical and visual ability in frontotemporal dementia* [38], *Art and the brain: The influence of frontotemporal dementia on an accomplished artist* [37], *Portraits of Artists. Emergence of Visual Creativity in Dementia* [39]. All these papers focus on the disease and cognitive processes in relation to the brain associated with artistic and creative experiences, in fact, the most relevant paper of the cluster [10] is specifically oriented to the influence of neuroscience and neuropharmacology in mental and physical health, and even considers the associated ethical issues.

## Discussion

There is a trend that goes from a more quantitative analysis of the brain in relation to creativity and personality to studies that give more importance to a qualitative approach that focuses more on humanistic topics such as aesthetics, emotions, perception, beauty, affect, consciousness or empathy. Indeed, Fig. 4 reveals that the keywords of the early period were more oriented to the basic understanding of the brain (e.g., brain, brain atlas, anatomy), which is logical because, given the technological advances of the time, the scientific community was more interested in discovering the basic functions of the brain as an object of study in itself. Thus, the practical applications are very specific, not including the arts and humanities as disciplines, but focusing more on artistic creativity and drawing as a personality trait, investigating disease in specific individuals and their cognitive processes, with a considerable presence of fMRI techniques. In contrast, after 2008, the trends of keywords such as arts, humanities, aesthetics or neuroaesthetics start to consolidate; terms that come to occupy the main clusters of the basic and motor themes in Fig. 5. Meanwhile there is also an increase in the use of EEG techniques, together with machine learning and deep learning becoming trends in the last years. Of course, there are still terms related to personality trauma and neurodegenerative diseases such as dementia or Alzheimer; however, what is striking in the current analysis is how these studies are framed within a broader approach in which the artistic and humanistic disciplines guide most of the research.

Certainly, the term neuroaesthetics as a neologism reflects very well this convergence of research and is a term that has significantly increased its appearance over time. A few years ago, Chatterjee [11] assessed the history of neuroaesthetics and its development as a discipline, proposing that it constitutes an emerging discipline that can be applied to very diverse fields, from explorations of human behavior when performing an instinctive activity (such as mate selection) to more social and sophisticated activities (such as consumption, communication or art). For Chatterjee, neuroethics is a promising field of study that aspires to become a mainstream science. The current analysis not only confirms this judgment, but also reaffirms something that for Chatterjee did not seem so clear,the predominance of the application of the term specifically in the arts and humanities in recent years.

More recently, Skov & Nadal (2023) state in *The Routledge International Handbook of Neuroaesthetics* that the term was born in 2000, but only started to be used frequently from 2010 onwards. Such an observation is confirmed in Figs. 4 and 5. In the latter, the term appears with a very high frequency in the second period (2009 to 2018), constituting a basic theme, and even becomes much more relevant in the third period of the map (2019 to 2022), when it is classified as a motor theme. However, Skov himself has made another very significant contribution to the subject (Anglada-Tort & Skov, 2020), when he states that the term aesthetics, despite being used more in all kinds of sciences, its use lacks unity of common approach. These authors propose to distinguish between aesthetics when it refers to sensible experience and aesthetics when it refers to the experience of art. The present study can qualify that observation, adding that in the specific case of neuroaesthetics, there is a growing tendency to study it within the framework of the convergence of neurosciences with the arts and humanities; despite the resistance of these fields to include foreign approaches [48].

Another term that has been gaining importance across the years is that of neuroethics, a motor theme in the second period of Fig. 5 and a keyword with considerable centrality and directly connected to humanities in Fig. 6*.* This field is of utmost importance in the development of future progress in neuroscience, since the Big Data era is catching up with almost all fields [21]. Neuroscience is no exception, since the access to servers and databases with neurological data can improve the training of robust and efficient machine learning and deep learning algorithms for the identification of patterns in neurological problems. Examples of the use of deep learning for neuroscience has led to the development of algorithms for epilepsy detection, prediction, emotion recognition, and mental state identification [1, 44]. However, strong ethical justifications and protocols need to be implemented and followed when working with a large amount of personal data from patients, therefore the importance of a global, transdisciplinary, and integrated community of researchers to address these challenges (Emerging Issues Task Force, International Neuroethics Society, 2019).

## Conclusions

The current study aimed at identifying the specific relationship between neuroscientific approaches and artistic and humanistic experiences by evaluating century-long scientific production in terms of main historical periods, conceptual structure and intellectual structure. The main findings were: a) the quantitative and neuroanatomical approach to creativity as a personal trait has been enriched with qualitative aspects, taking into consideration more humanistic topics such as art, perception, aesthetics and beauty in healthy individuals; b) the use of fMRI has been slightly displaced in recent years by EEG techniques, a displacement that has been accompanied by the emergence of new machine learning and artificial intelligence models, which resort to biometric measurement and deep learning, more flexible data models that better deal with the qualitative aspects of the arts and humanities; c) four main clusters were identified in the study: humanistic, creative, neuroaesthetic, and medical; neuroaesthetics cluster, despite being the smallest, is the most relevant, having more centrality, and mediating the convergence of the clusters based on creativity and neuroscience; d) the clustering of the network of authors confirms the central relevance of neuroscientific studies on the subjective value of art and beauty, with an important presence of fMRI technologies, separated from another cluster dedicated to the cognitive process and a smaller one focused on the relationship between the experience of art and mental illness; e) neuroaesthetics is not the only neologism appearing in this converging field of study, but also neuroethics, due to the strong protocols and the storage of large personal data sets involved in the applications of new machine learning and deep learning models.

In general, the abundance of this type of research corroborates the fertility of neuroscientific approaches, in this case applied to the arts and humanities. Everything indicates that the interest in this area of study will be greater in the coming years, taking into account the recent involvement of such innovative technologies with so much potential such as machine learning and deep learning. The current study suggests that the arts and humanities will not be a field of study alien to these technological innovations, but on the contrary, a field that can take advantage of them despite the complexity and subjectivity that affects their objects of study.

There are methodological limitations in the bibliometric approach, since it sometimes does not allow the deserved depth of analysis, but rather gives rise to interpretations that should always be corroborated by closer reading of the references. Another technical limitation is the selection of a single database as a source of information, since despite the breadth of references collected in Scopus, there are other databases that can complement the research. Further in relation to the analyses, it is worth mentioning that the division by historical periods established, although it has been carried out based on the data obtained, it could be decided to do otherwise, or even to make a more exhaustive division, which would result in a more precise, but also broader study, which goes beyond the limits of this study. Thus, future studies could address in more detail some specific period. Another possibility for new research complementary to the present one, which is more conceptual, is that of the evolution and incidence of material innovations, more particularly, how portable neurotechnologies have been making their way into the context of art and the humanities.

## Data Availability

https://doi.org/10.6084/m9.figshare.23929893.

## Abbreviations

EEG: Electroencephalogram
fMRI: Functional magnetic resonance imaging
RQ: Research question
